# cAMP-PKA/EPAC signaling and cancer: the interplay in tumor microenvironment

**DOI:** 10.1186/s13045-024-01524-x

**Published:** 2024-01-17

**Authors:** Hongying Zhang, Yongliang Liu, Jieya Liu, Jinzhu Chen, Jiao Wang, Hui Hua, Yangfu Jiang

**Affiliations:** 1 Cancer Center, Laboratory of Oncogene, State Key Laboratory of Biotherapy, West China Hospital, Sichuan University, Chengdu, 610041 China; 2 School of Basic Medicine, Chengdu University of Traditional Chinese Medicine, Chengdu, 610075 China; 3 Laboratory of Stem Cell Biology, West China Hospital, Sichuan University, Chengdu, 610041 China

**Keywords:** cAMP, cAMP-dependent protein kinase, Cancer, Exchange protein activated by cAMP, Immunotherapy, PKA, Tumor microenvironment

## Abstract

Cancer is a complex disease resulting from abnormal cell growth that is induced by a number of genetic and environmental factors. The tumor microenvironment (TME), which involves extracellular matrix, cancer-associated fibroblasts (CAF), tumor-infiltrating immune cells and angiogenesis, plays a critical role in tumor progression. Cyclic adenosine monophosphate (cAMP) is a second messenger that has pleiotropic effects on the TME. The downstream effectors of cAMP include cAMP-dependent protein kinase (PKA), exchange protein activated by cAMP (EPAC) and ion channels. While cAMP can activate PKA or EPAC and promote cancer cell growth, it can also inhibit cell proliferation and survival in context- and cancer type-dependent manner. Tumor-associated stromal cells, such as CAF and immune cells, can release cytokines and growth factors that either stimulate or inhibit cAMP production within the TME. Recent studies have shown that targeting cAMP signaling in the TME has therapeutic benefits in cancer. Small-molecule agents that inhibit adenylate cyclase and PKA have been shown to inhibit tumor growth. In addition, cAMP-elevating agents, such as forskolin, can not only induce cancer cell death, but also directly inhibit cell proliferation in some cancer types. In this review, we summarize current understanding of cAMP signaling in cancer biology and immunology and discuss the basis for its context-dependent dual role in oncogenesis. Understanding the precise mechanisms by which cAMP and the TME interact in cancer will be critical for the development of effective therapies. Future studies aimed at investigating the cAMP-cancer axis and its regulation in the TME may provide new insights into the underlying mechanisms of tumorigenesis and lead to the development of novel therapeutic strategies.

## Introduction

Cyclic adenosine monophosphate (cAMP) is a signaling messenger derived from the hydrosis of ATP by the transmembrane or soluble adenylate cyclase (AC). The downstream effectors of cAMP include cAMP-dependent protein kinase (PKA), exchange protein activated by cAMP (EPAC) and ion channels. The first cDNA encoding the catalytic subunits of PKA was isolated in 1986 [1]. Thereafter, more isoforms of the catalytic subunit of PKA were cloned. cAMP-responsive element-binding protein (CREB) is one of the classical effectors of cAMP-PKA/EPAC pathways. As a well-known second messenger, cAMP can regulate cell differentiation, proliferation, survival and migration. Thus, cAMP has pleiotropic effects on both physiological and pathophysiological processes, such as development, reproduction, angiogenesis, cell cycle progression, insulin secretion, energy metabolism, and pluripotent stem cell reprogramming.  Besides, nuclear cAMP signaling is important for dopaminergic neurotransmission, learning and memory [2, 3].

Aberrant cAMP signaling may lead to many diseases such as cardiomyopathy, cognitive impairment, oncogenic virus pathogenicity, and autoimmune diseases including multiple sclerosis, rheumatoid arthritis, inflammatory bowel disease and systemic lupus erythematosus [4]. During the latent stage of Epstein-Barr virus (EBV) infection, the cAMP/PKA pathway promotes the stimulation of viral proteins expression by EBNA2, an EBV-encoded protein, and thereby enhances viral persistence and EBV-associated oncogenesis [5]. In addition, cAMP may stimulate HIV-1 transcription in latently infected monocytes/macrophages and reverse HIV latency [6]. However, some isoforms of cAMP responsive element modulator (CREM, also called inducible cAMP early repressor), one of the PKA targets, can inhibit HIV long terminal repeat promoter activity thereby suppressing HIV infection [7]. Moreover, cAMP can suppress the progression of autoimmune diseases by preventing the formation of neutrophil extracellular traps, the activation of NF-κB, the production of inflammatory cytokines such as tumor necrosis factor, IL1β and IL6, T cell activation, and autoantibody production [8–10].

Since cAMP signaling regulates cell proliferation and differentiation, abnormal cAMP biogenesis is involved in tumorigenesis, a complex disease resulting from diverse environmental and genetic factors [11]. cAMP has a significant impact on both cancer cells and the tumor microenvironment (TME). Cytokines, hormones and growth factors can modulate cAMP signaling by engaging the generation of cAMP from ATP and the transduction of signals by protein kinases. On the other hand, tumor-associated stromal cells, such as fibroblasts, endothelial cells and immune cells, can produce and release signaling molecules that either stimulate or inhibit cAMP synthesis. cAMP not only directly regulates cancer cell proliferation, survival and migration, but also promotes immune evasion by reprogramming the TME.

Notably, cAMP has paradoxical effects on tumorigenesis, depending on the cancer types, stages and other contexts. Thus, both the antagonists and agonists of cAMP signaling can be exploited for cancer treatment. Although the structure of PKA catalytic subunit has been solved more than 30 years ago [12], there are few PKA inhibitors available in the market. No PKA inhibitors have been approved in the clinic. However, phosphodiesterases (PDEs) inhibitors, which prevent cAMP or cGMP hydrolysis and thereby elevate cAMP/cGMP levels, are developed for the treatment of obstructive respiratory diseases, neuroinflammation, ischemia/reperfusion injury, atopic dermatitis, erectile dysfunction, depression and hypertension [13–15]. Preclinical studies demonstrate that PDE inhibitors may have tumor-suppressive effects, especially when combined with chemotherapeutic agents [16, 17]. The present review updates recent advances in cAMP signaling, and discusses the complex roles of cAMP signaling in the TME. Considering the implication of cAMP signaling in cancer biology, the potential to selectively target PKA, EPAC or PDEs, and the possibility of either elevating or reducing cAMP levels in the TME, targeting cAMP signaling pathways holds promise in cancer therapy.

## Overview of the cAMP signaling pathways

**Fig. 1:**
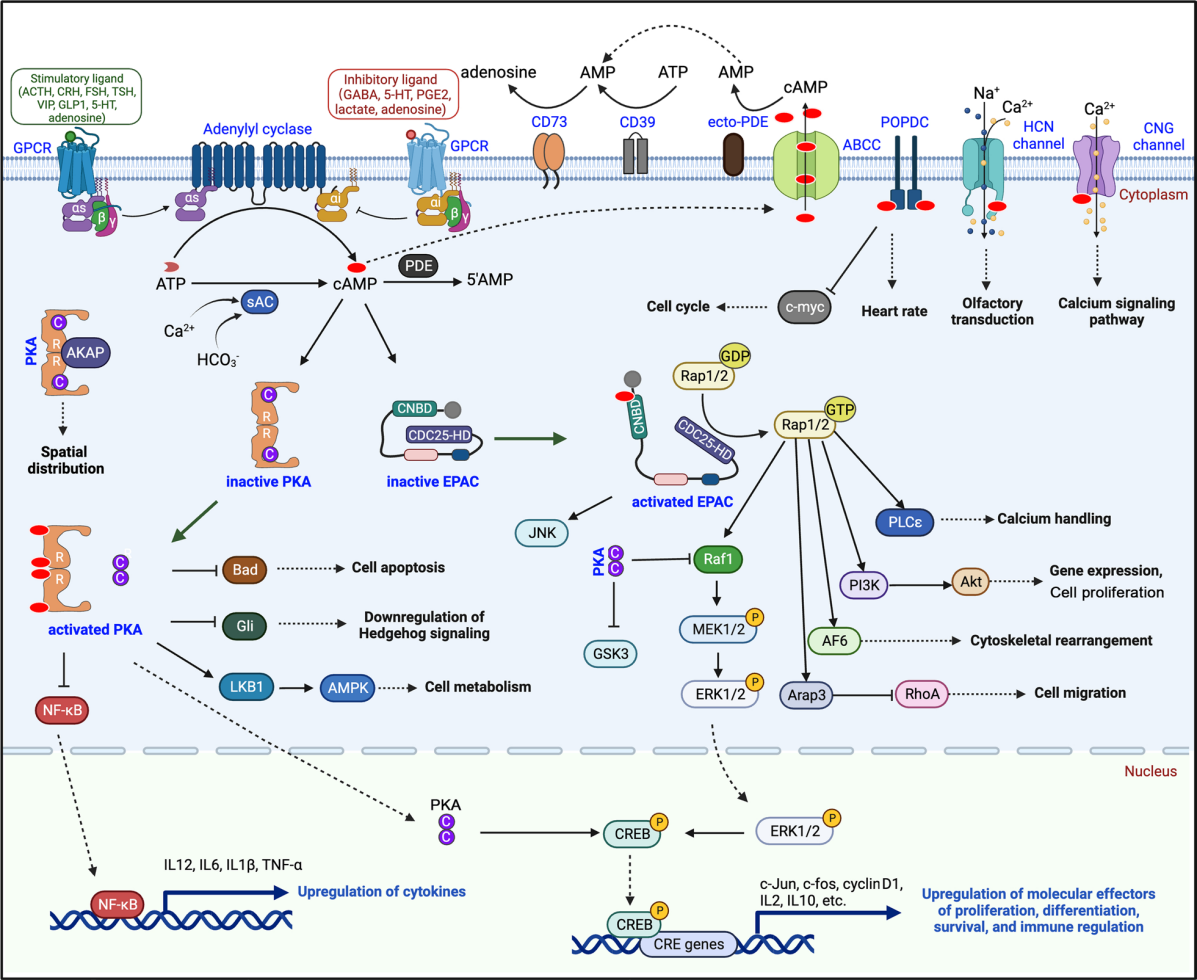
The cAMP signaling pathways. The extracellular cAMP can be converted into AMP by plasma membrane-anchored ecto-PDE, followed by the conversion of AMP into adenosine by CD73. Ligands-engaged Gs- or Gi-coupled receptors may stimulate or inhibit cAMP synthesis, respectively. The ABCC transporter family members ABCC4/5/11 mediate the export of cAMP to extracellular space. cAMP engages PKA, EPAC, POPDC, HCN and CNG channels to regulate diverse signaling pathways and the transcription activities

In mammals, there are nine adenylate cyclase genes (AC1-9) encoding transmembrane adenylate cyclases. These transmembrane adenylate cyclases have distinct physiological activities, which are generally regulated by heterotrimeric G proteins upon activation of G protein-coupled receptors (GPCRs) by extracellular hormones (adrenocorticotropic hormone, corticotropin-releasing hormone, follicle-stimulating hormone and thyroid stimulating hormone) [18], neurotransmitters (catecholamines, vasoactive intestinal peptide, glucagon-like peptide 1, gamma-aminobutyric acid, serotonin, etc.), cytokines (prostaglandin E2, netrin-1), and physiological agents such as adenosine and lactate [19, 20] (Fig. 1). Upon stimulation, G proteins dissociate into free Gα and Gβγ subunits. Gαs-coupled GPCRs stimulate adenylate cyclase activity and cAMP synthesis, while Gαi-coupled GPCRs inhibit type I, V and VI adenylate cyclase activation [21]. Except for the plasma membrane-initiated cAMP synthesis, the stimuli-induced internalization of GPCRs to endosomes and/or the trans-Golgi network leads to sustained generation of cAMP. On the other hand, cytosolic or nuclear translocated sAC (soluble adenylate cyclase) can be directly activated by HCO_3_^−^ or Ca^2+^, and catalyze cAMP synthesis [22]. The transmembrane adenylate cyclases and sAC co-operatively or independently build cAMP pools in the cytosol, mitochondria and nucleus, allowing compartmentalized cAMP signaling [23]. cAMP signaling is transduced by at least five protein families, including PKA, EPAC, cyclic nucleotide-gated channels and hyperpolarization-activated cyclic nucleotide-gated channels (CNG/HCN), Popeye domain containing protein (POPDC), and cyclic nucleotide receptor involved in sperm function (CRIS). Both PKA and EPAC are widely expressed in many tissues and cancers.

### The cAMP signal transducer PKA

The spatiotemporal propagation of cAMP is critical for appropriate response to various stimuli. PKA is one of the classical cAMP effectors. Both cAMP-synthesizing enzymes and the cAMP effector proteins are confined to intracellular nanodomains that contain distinct isoforms of adenylate cyclase, A kinase anchor proteins (AKAPs) and PDEs. Different AKAPs anchor PKA to distinct sites and thereby enable PKA to phosphorylate specific targets. On the other hand, AKAPs may either positively or negatively regulate adenylate cyclase activity, depending on the types of these enzymes [24]. AKAPs, PDEs and other regulators contribute to local cAMP gradients. PDEs comprise a family of enzymes that catalyze the degradation of cAMP and cGMP to AMP and GMP, respectively. Among the 11 families (PDE1–11) and over 100 estimated isoforms, PDE4/7/8 are specific to cAMP, while PDE5/6/9 selectively hydrolyze cGMP [25]. The other PDEs, including PDE1/2/3/10/11, can degrade both cAMP and cGMP [26–28]. The intracellular concentration and distribution of cAMP is determined by the balance between the activities of PDEs and adenylate cyclase.

cAMP not only elicits intracellular signaling transduction, but also releases into extracellular space and the bloodstream. The ABCC transporter family members ABCC4/5/11 mediate the export of cAMP to extracellular space [29]. In turn, the extracellular cAMP is converted into adenosine by ectonucleotide pyrophosphatase/phosphodiesterase 1 (ecto-PDE/ENPP1) and ectonucleotide 5′-nucleotidase (NT5E/CD73) [30, 31]. Finally, adenosine receptors mediate the paracrine action of cAMP in different tissues. While secreted cAMP promotes directional chemotaxis via cAMP receptors in bacteria such as Dictyostelium discoideum [32], it remains to know whether the plasma membrane cAMP receptors are present in mammalian cells.

**Fig. 2:**
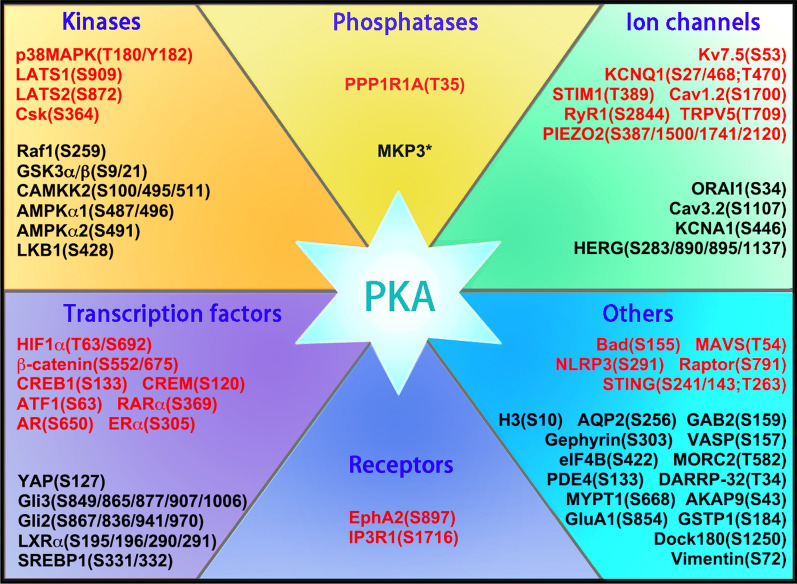
Representative substrates of PKA. PKA can phosphorylate a number of proteins including kinases, phosphatases, transcription factors, receptors, ion channels, etc. Phosphorylation of these substrates by PKA may either enhance (red font) or inhibit (black font) their activities. The phosphorylation sites in these substrates are shown. *, The phosphorylation site is undefined

As a cAMP-dependent protein kinase, PKA assembles as a tetramer consisting of two catalytic (C) subunits and two regulatory (R) subunits [25]. Upon four molecules of cAMP binding to PKA regulatory subunits, the tetramer dissociates into two free C subunits and a R subunit dimer, resulting in PKA activation. In addition, TGFβ-activated SMAD3/4 can interact with PKA regulatory subunits and promote PKA activation [33]. On the other hand, cAMP can enhance TGFβ signaling in breast cancer cells by inducing TGFβ receptor I expression [34]. Therefore, the cAMP and TGFβ signaling pathways are interconnected. Active PKA phosphorylates many proteins including protein kinases, phosphatases, transcription factors, receptor proteins, ion channels and cytoskeleton proteins (Fig. 2). PKA-induced phosphorylation of its targets either enhances or inhibits their activity. CREB is a family of transcription factors that comprises CREB1, CREM and activating transcription factor 1 (ATF-1) [35]. These transcription factors can bind to cAMP response element in the promoter of many genes, including c-Jun, c-Fos, cyclin D1, IL2, and IL10. Phosphorylation of CREB/ATF1 by PKA enhances their transcriptional activity. In contrast, phosphorylation of GSK3 and Raf1 by PKA leads to their inactivation [36, 37]. Furthermore, the mitochondrial AKAP1 and PKA can prevent mitochondrial dysfunction [38]. Since mitochondria are essential for tumor progression [39], the reliance of PKA to relieve mitochondrial stress in cancer warrants further studies.

The specificity of PKA is largely determined by AKAPs, which deliver PKA to distinct signosomes. AKAPs are a family of functionally related proteins with diverse structure that comprise more than 60 members [40]. The PKA regulatory subunits interact with a 14–18 residue amphipathic helix in AKAPs through the docking and dimerization (D/D) domain. AKAPs serve as the scaffolding proteins to assemble signaling complexes containing other molecules such as phosphatases, kinase, adenylate cyclase and PDEs [41]. Tethering by AKAPs is a pivotal determinant in dictating which PKA substrates become phosphorylated in different cell types and contexts. Most cells express between 10 and 15 different AKAPs [42]. Seven AKAPs have been identified in lipid rafts in T cells and have been shown to contribute to the maintenance of T cell homeostasis [43].

**Fig. 3:**
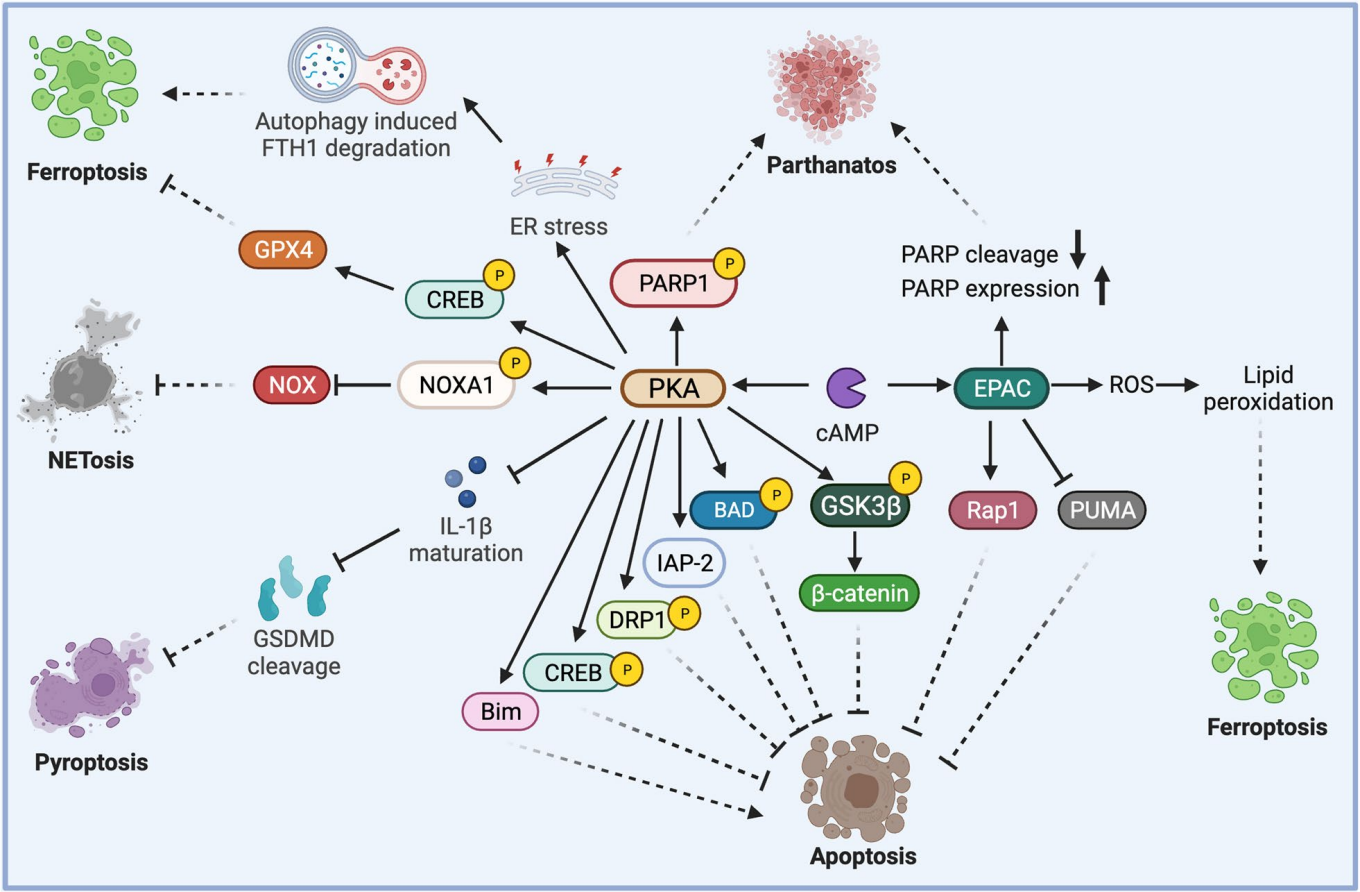
The effects of cAMP signaling on programmed cell death. PKA can regulate apoptosis, pyroptosis, ferroptosis, parthanatos and NETosis. EPAC also regulates apoptosis, ferroptosis and parthanatos

Programmed cell death is an important biological process for physiological homeostasis, tissue renewal, and tumorigenesis. Many targets of PKA are involved in programmed cell death (Fig. 3). PKA phosphorylates and inactivates GSK3 and BAD, thereby inhibiting apoptosis [44]. However, cAMP-PKA also induces the proapoptotic B-cell lymphoma-family protein Bim and promotes the apoptosis of some cell types [45]. In addition, PKA suppresses IL1 maturation and thereby prevents gasdermin D (GSDMD) cleavage and pyroptosis [46]. Phosphorylation of poly(ADP-ribose) polymerase 1 (PARP1) by PKA leads to mitochondrial and nuclear PARP1 activation, thus impairing the function of mitochondria and inducing parthanatos [47]. Also, cAMP may inhibit NETosis, a type of neutrophil cell death associated with the release of neutrophil extracellular traps (NETs) [48]. Ferroptosis is another type of programmed cell death triggered by labile iron and lipid peroxidation. PKA had both pro-ferroptosis and anti-ferroptosis effects. Phosphorylation of CREB by PKA induces GPX4 expression, which antagonizes lipid peroxidation and then inhibits ferroptosis. On the other hand, cAMP-PKA signaling may induce ER stress, leading to autophagy-induced degradation of ferritin heavy chain 1, intracellular accumulation of Fe^2+^, and ferroptosis [49]. However, recent studies indicate that PKA may inhibit ER stress and ferroptosis as well [50]. Thus, the effects of cAMP and PKA on ferroptosis may be context-dependent.

### The cAMP sensor EPAC

EPACs are the cAMP-regulated guanine nucleotide exchange factors that mediate numerous responses to cAMP [51]. Unlike PKA, EPAC proteins are single chain multi-domain polypeptides that contain both catalytic and regulatory elements. Both EPAC1 and EPAC2 consist of a C-terminal catalytic region and an auto-inhibitory N-terminal regulatory region. EPAC2, however, has an additional N-terminal cyclic nucleotide-binding domain [52]. The catalytic region consists of a Ras-exchange motif domain (REM), a Ras-association domain, and a cell division cycle 25 (CDC25) homology domain.

Binding of cAMP to the cAMP-binding domain in the regulatory region of EPAC results in conformational changes and the activation of Ras-related proteins Rap1 and Rap2. G protein-coupled receptor kinase 2 (GRK2) negatively regulates EPAC-mediated Rap1 activation by phosphorylating EPAC1 at Ser-108 [53]. The activation of PLCε by Rap1 in turn activates protein kinase C (PKC). Of note, both PKA and EPAC may mediate the cAMP‐induced activation of PKCε [54]. EPAC-PKC signaling promotes the phosphorylation of mitochondrial enzyme pyruvate dehydrogenase (PDHA1) and thereby enhances mitochondrial respiration [55]. Meanwhile, both PKA and EPAC-mediated activation of PKCε promote phospholamban phosphorylation at Ser-16, which contributes to calcium homeostasis, cardioprotection and germ cell differentiation [56]. In addition, activation of PKC by EPAC1-Rap1 signaling may sensitize TRPV1- and Piezo2-mediated mechanotransduction, which is involved in inflammatory pain and tumor progression [53, 57]. The stimulation of EPAC/Rap pathway by cAMP may enhance PI3K/Akt/mTOR and Raf/Ras/MAPK signaling, which are implicated in various physiological and pathophysiological processes such as cell growth, migration, adhesion and spreading [58, 59]. Except for the guanine nucleotide exchange factor activity of CDC25 homology domain, the REM domain of EPAC is able to induce JNK activation [60].

Similar to PKA, EPAC can regulates apoptosis, parthanatos and ferroptosis (Fig. 3). Activation of Rap1 and inhibition of the pro-apoptotic PUMA by EPAC suppresses apoptosis [61, 62]. In contrast, EPAC promotes ROS-mediated lipid peroxidation and ferroptosis [63]. EPAC also promotes PARP1 expression and inhibits PARP1 cleavage, thereby inducing parthanatos [64]. Thus, EPAC generally inhibits apoptosis, but paradoxically promotes parthanatos and ferroptosis. The effects of EPAC on cell death may be dependent on the stimuli.

### Cyclic nucleotide-activated ion channels and POPDC proteins

Cyclic nucleotide-activated ion channels play a fundamental role in a variety of physiological processes. Hyperpolarization-activated cyclic nucleotide-gated channels (HCN) belong to the superfamily of voltage-gated pore loop channels. HCN channels regulate electrical activity in the excitable brain and heart cells [65]. Voltage-dependent opening of these channels is directly regulated by cAMP, contributing to spontaneous rhythmic activity in both brain and heart [65]. The unliganded cyclic nucleotide binding (CNB) fold in the C-terminal region of HCN channels and the transmembrane region co-operatively autoinhibit channel activity, while cAMP binding to the CNB fold relieves the autoinhibition [65, 66]. In addition, cyclic nucleotide-gated (CNG) channels are non-selective cation channels that play a crucial role in visual and olfactory signal transduction [67].

POPDC proteins are three-pass transmembrane proteins with high affinity to bind cAMP [68]. The Popeye domain of POPDC is supposed to undergo a conformational change upon cAMP binding. POPDC are important players in cardiac and skeletal muscle physiology. POPDC1 (also called BVES) serves as an adaptor for the interaction between adenylate cyclase 9 and TREK-1 potassium channels that control heart rate in a cAMP-dependent manner [69]. Similar to that PKA and EPAC exist in complexes containing PDEs, POPDC proteins are found in complexes with PDE4, which ensures an optimal cycle length of Ca^2+^ transients firing in sinoatrial nodes [70]. In addition, POPDC1 and POPDC3 expression is decreased in breast, brain, colon and gastric cancers [71–73]. The frequent silencing of POPDC in cancer may be attributed to promoter hypermethylation [74]. EGFR also downregulates POPDC1 expression in breast cancer [72]. The decrease in POPDC expression correlates with enhanced tumor progression and drug resistance, and poor patient survival, suggesting that POPDC is a tumor suppressor [68]. POPDC1 interacts with the tight junction protein ZO-1 and vesicle-associated membrane protein 3, thereby maintaining epithelial integrity and the recycling of transferrin, transferrin receptor, and integrin [75, 76]. Furthermore, POPDC1 negatively regulates RhoA activity and the expression of several oncogenes including c-myc, β-catenin and MMP2/9, thereby suppressing epithelial-mesenchymal transition (EMT) [77, 78]. Therefore, POPDC may be potential target for cancer therapy.

## Contextual roles of cAMP signaling in cancer cells

Accumulating evidence demonstrates that cAMP signaling has paradoxical effects in different types of cancer (Table 1). While the targets of PKA include both oncogene and tumor suppressors, POPDCs are largely tumor suppressive. Therefore, the net effects of cAMP on tumor progression may depend on the balance between tumor-promoting and tumor-suppressing effects. In many types of cancer, cAMP signaling exhibits tumor-promoting properties [35]. The activation of cAMP-mediated PKA and EPAC signaling have been shown to promote tumor cell proliferation, survival, motility, adhesiveness and invasiveness in preclinical models of liver, lung, breast, brain and lymph cancers [35, 61]. PKA is deregulated in several cancers, especially in endocrine tumors such as adrenal tumors (Carney complex, Cushing’s syndrome), thyroid cancer, and growth hormone-secreting pituitary tumors [79–83]. Germline mutations in *PRKAR1A* cause Carney Complex, a disorder manifested as adrenal cortex hyperplasia/adenomas, cardiac and other myxomas, spotty skin pigmentation, and other abnormalities [84]. The majority of *PRKAR1A* mutations lead to decreased *PRKAR1A* mRNA due to nonsense-meditated mRNA decay, thereby reducing the levels of this regulatory subunit and increasing PKA activity [85]. Somatic mutations in *PRKACA* and *PRKACB* are also identified as driver mutations in adrenal cortical adenoma [86, 87]. In addition, mutations in *PRKACA*, *PRKACB* and *PRKAR1A* have been detected in liver, stomach, pancreas, lung and ovary cancers. The fusion of *PRKACA* and *DNAJB1* is dominant and oncogenic in fibrolamellar hepatocellular carcinoma (FLC) [88], while the *DNAJB1-PRKACA* or *ATP1B1-PRKACB* fusion is also detected in pancreatobiliary neoplasms [89, 90].

**Table 1:** The roles of cAMP signaling in cancer

Cancer type	Effects of cAMP signaling	References
Leukemia	cAMP promotes leukemia progression	[91]
	cAMP inhibits anthracycline- and DNA-damage-induced apoptosis	[92]
	cAMP-EPAC promotes ribosome-targeting therapy resistance in AML	[93]
	cAMP sensitizes T-ALL to dexamethasone	[94]
	cAMP-PKA sensitizes AML to GSKJ4	[95]
Diffuse large B cell lymphoma	cAMP inhibits tumor cell survival and drug resistance	[96]
Lung cancer	cAMP-PKA promotes cancer cell survival and EGFR inhibitor resistance	[97, 98]
	CREB1 enhances cisplatin sensitivity in lung cancer cells	[99]
Gastric cancer	cAMP promotes gastric carcinogenesis through activation of CREB, EPAC and DARPP-32	[100–104]
Liver cancer	PKA activation drives fibrolamellar liver carcinoma	[105, 106]
	GNAS mutation promotes liver carcinogenesis via cAMP/JAK/STAT3 signaling	[107]
	CREB promotes HCC metastasis and drug resistance	[108–110]
	cAMP/PKA mediates the tumor-suppression effects of Ex-4	[111]
	PDE4 inhibition suppresses HCC cell proliferation and survival	[112, 113]
Colorectal cancer	GPR43 deficiency promotes colon carcinogenesis by upregulated cAMP/PKA signaling	[114]
	Norepinephrine promotes colon cancer cell growth and invasion by inducing CREB phosphorylation	[115]
Breast cancer	PKA phosphorylates ERα and promotes tamoxifen resistance	[116]
	cAMP/PKA promotes chemotherapeutic resistance in inflammatory breast carcinoma	[117]
	PAQR8 downregulates cAMP levels and promotes drug resistance	[118]
	PKA upregulates PTEN and p53 and inhibits cell growth	[119, 120]
	PKA enhances doxorubicin sensitivity in TNBC	[121]
	cAMP/PKA inhibits NF-κB and breast cancer stemness	[122]
Ovarian cancer	cAMP inhibits p53 and DNA-damage-induces apoptosis in BRCA1-deficient ovarian cancer	[123]
	cAMP inhibits JNK activity and apoptosis	[124]
Melanoma	cAMP-EPAC inhibits PUMA expression and apoptosis	[62]
	cAMP stimulates the growth of primary melanoma but not the metastatic melanoma	[125–127]
	cAMP-PKA promotes vemurafenib resistance	[128]
Prostate cancer	PKA promotes AR activation, and abiraterone, enzalutamide, castration resistance	[129–132]
	cAMP-PKA promotes metastasis	[133]
Medulloblastoma	cAMP-PKA suppresses medulloblastoma by phosphorylating and inactivating Gli	[134]
Basal cell carcinoma of the skin	cAMP-PKA abolishes oncogenic Sonic hedgehog signaling and suppresses tumor growth	[135]

As one of the targets of PKA, CREB promotes cancer cell proliferation, survival and migration, and negatively associates with cancer patients survival [136]. cAMP also induces CREB3L1 expression through PKA-p38MAPK pathway [137]. While CREB3L1 promotes the growth and metastasis of anaplastic thyroid carcinoma, it inhibits bladder cancer metastasis and glioblastoma growth [138–140]. Contradictory effect of CREB3L1 on triple negative breast cancer has been reported. One study shows that CREB3L1 promotes triple negative breast cancer metastasis [141], whereas another study demonstrates that CREB3L1 inhibits triple negative breast cancer metastasis [142]. The reason for such discrepancy is unclear. Given the versatile effects of diverse cAMP-regulated proteins on cancer, it is not surprising that cAMP may promote the progression of some types of human cancer but inhibit other cancer types. A more fascinating fact is that cAMP can even exert opposing effects on the same type of cancer in a context-specific manner. PKA is one of the dependencies in cancers such as fibrolamellar carcinoma and chondroma [143], while it is a bona-fide tumor suppressor in medulloblastoma.

### Roles of cAMP in leukemia and lymphoma

cAMP promotes leukemia progression and inhibits anthracycline-, DNA damage- and ribosome biogenesis inhibitor-induced apoptosis in leukemia cells [91–93]. The PKA substrate CREB can promote hematopoietic cell proliferation and myeloproliferative disease [144, 145]. Overexpression of CREB and increased CREB phosphorylation are detected in the majority of acute myeloid leukemia patients, which is associated with poor prognosis in these patients [146]. Microsomal prostaglandin E synthase-1 also induces the expression of metadherin through the PGE2/EP3/cAMP/PKA/CREB pathway and thereby promotes T-cell acute lymphoblastic leukemia progression [147]. In addition, EPAC1/2 overexpression is detected in human acute myeloid leukemia. Inhibition of EPAC1/2 can suppress AML cell survival [93]. The cAMP‐EPAC1/2‐Rap1 survival pathway also contributes to ribosome‐targeting therapy resistance in patients with acute myeloid leukemia [93]. In addition, autophagy, a lysosome-dependent catabolic pathway, is involved in tumor progression and therapy [148]. cAMP signaling may promote PARP1-mediated autophagy and thereby inhibit DNA damage-induced B-cell precursor acute lymphoblastic leukemia cell death [149, 150].

However, cAMP signaling is required for the induction of gene expression by dexamethasone, a popular antileukemia agent [94, 151]. The expression of EP4, a PGE2 receptor, in T-cell acute lymphoblastic leukemia samples leads to an increase in intracellular cAMP levels, which sensitizes T-ALL to dexamethasone. In contrast, downregulation of cAMP synthesis and signaling confers dexamethasone resistance [94]. Moreover, combination of all-trans retinoic acid and arsenic trioxide can successfully treat patients with low-risk acute promyelocytic leukemia [152]. The induction of cAMP/PKA pathway contributes to retinoic acid-induced acute promyelocytic leukemia cell differentiation [153]. Downregulation of adenylate cyclase may reduce intracellular cAMP levels and suppress the induction of acute promyelocytic leukemia cell differentiation by all-trans retinoic acid [154, 155]. Stimulation of cAMP-PKA pathway by forskolin also sensitizes acute myeloid leukemia cells to GSKJ4, a H3K27me2/3 demethylases inhibitor [95].

In diffuse large B-cell lymphoma, cAMP inhibits cell growth in PKA- and EPAC-independent manner. The inhibition of PI3K/Akt pathway contributes to the effect of cAMP on diffuse large B cell lymphoma cell. PDE4B, a main hydrolyzer of cAMP in B cells, promotes diffuse large B-cell lymphoma cell survival and drug resistance by downregulation of cAMP signaling [96]. It is possible that the inhibition of PI3K/Akt by cAMP outweighs other potential tumor-promoting effects of cAMP in diffuse large B-cell lymphoma.

### Roles of cAMP in solid tumors

cAMP signaling plays complex roles in various types of solid tumors. Here, we provide an update about the functions of cAMP in lung, liver, stomach, colon, breast, prostate and brain cancer, as well as melanoma. For the findings on cAMP signaling in ovarian cancer, cholangiocarcinoma and thyroid cancer, interested readers can refer to related reviews [82, 156, 157].

#### Lung cancer

Inactivating mutation of the tumor suppressor *STK11/LKB1* is one of the genomic drivers of KRAS-mutated lung adenocarcinoma. LKB1 may inhibit the nuclear translocation of CREB-regulated transcription coactivators and thereby suppress constitutive activation of cAMP/CREB-mediated transcription [158]. Therefore, inactivating *LKB1* mutation reinforces the cAMP-PKA-CREB signaling in lung cancer cells [158]. Reciprocally, PKA phosphorylates human and murine LKB1 at Ser-428 and Ser-431, respectively [159]. Phosphorylation of LKB1 at this residue suppresses the activation of AMPK and abrogates the tumor suppressive effects of LKB1 on melanoma [160], while it remains unclear whether the same is true for lung cancer. Of note, PKA can directly phosphorylate and inactivate AMPK, indicating that PKA may inhibit AMPK even in LKB1-mutated cancer [161]. In addition, CREB suppresses lipid peroxidation in lung adenocarcinoma cells by inducing *GPX4* transcription and then inhibiting ferroptosis [97]. However, CREB1 phosphorylation contributes to cisplatin sensitivity in lung cancer cells via regulation of the ERK pathway, especially in cancer cells with mutated SET domain containing 2 (SETD2), a histone H3 lysine 36 (H3K36) trimethyltransferase [99]. Except for CREB, the cAMP/PKA-regulated phosphoprotein DARPP-32 enhances ERBB3/EGFR heterodimerization and promotes EGFR inhibitor resistance in EGFR-mutated lung adenocarcinoma [98]. DARPP-32 also promotes small cell lung cancer growth [162]. Moreover, AKAP1 expression correlates with high levels of Myc, phosphorylated mTOR, and reduced lung cancer patients survival [163].

#### Digestive system cancer

Overexpression of CREB1 and EPAC1 is also associated with gastric cancer progression [100, 101]. In addition, DARPP-32 activates IGF1R and STAT3 signaling in gastric cancer cells [102]. NF-κB activation and hypermethylation-mediated silencing of miR490-3p by *H. pylori* lead to DARPP-32 overexpression in gastric cancer cells thereby activating PI3K/AKT and STAT3 signaling pathways [103, 104]. Therefore, *H. Pylori* infection and cAMP signaling may cooperatively promote gastric carcinogenesis. Since STAT3 is a potent oncogene in various types of cancer [164], the PKA-DARPP32-STAT3 axis may contribute to tumorigenesis at sites other than the stomach.

cAMP signaling has complex roles in liver tumorigenesis. While *DNAJB1-PRKACA* fusion drives oncogenic pathways and induces FLC [105], the mixed-FLC/HCC tumors with inactivating mutations or translocations in the gene encoding BRCA1-associated protein-1 (BAP1) also harbor a chromosome gain of *PRKACA* and a loss of the inhibitory regulatory subunit PRKAR2A, thus exhibiting addiction to PKA activation [106]. Moreover, GNAS-activating mutation promotes liver tumorigenesis by upregulating cAMP/JAK/STAT3 signaling [107]. Dopamine or PGE2 secretion in the tumor microenvironment also engages dopamine receptor D1 or EP4 receptor, respectively, to activate cAMP/CREB pathway and promote HCC progression [165, 166]. CREB activation promotes HCC metastasis and drug resistance [108–110]. However, cAMP/PKA mediates the tumor suppression effects of Exenatide (Ex-4), an antidiabetic drug targeting glucagon-like peptide-1 receptor, on HCC in obese diethylnitrosamine-treated mice [111]. Treatment of hepatoma cells with PDE4 inhibitors increases intracellular cAMP levels but suppresses cell cycle progression and survival [112]. PDE4D depletion also inhibits the expression of cancer-related genes such as IGF2 and the progression of cell cycle in HCC cells [113]. Together, these studies indicate that cAMP has opposing effects on HCC.

The cAMP-PKA-CREB pathway is frequently activated in colorectal cancer. Adenylate cyclase overexpression or PDE4B silencing may increase cAMP levels in colon cancer [167, 168]. In addition, the fermentation ofdietary fiber can generate short-chain fatty acids, which activate free fatty acid receptor 2 (FFAR2/GPR43) and reshape gut microbiota, leading to increased expression of inflammation suppressors and prevention of intestinal carcinogenesis [169]. In contrast, GPR43 deficiency results in enhanced cAMP-PKA-CREB signaling and HDAC expression, thereby promoting colon carcinogenesis by inhibiting the expression of inflammation suppressors and enhancing neutrophil infiltration into colon cancer [114]. Moreover, norepinephrine induces CREB phosphorylation and then promotes human colon cancer cells growth and invasion [115]. CREB crosstalks with KRAS to promote colon carcinogenesis and positively regulates ALCAM (CD166) and PROM1 (CD133) expression, thus promoting colorectal cancer stemness and metastasis [170, 171].

#### Breast cancer

What roles does cAMP play in breast cancer? About 60% of human breast cancer are estrogen receptor-positive. PKA directly phosphorylates estrogen receptor α, leading to ligand-independent estrogen receptor activation and tamoxifen resistance in estrogen receptor-positive breast cancer [116]. In addition, cAMP/PKA/CREB may act downstream of JAK/STAT3 to promote chemotherapeutic resistance in inflammatory breast carcinoma [117]. However, reduced cAMP levels contribute to the promotion of estrogen receptor inhibitor, HER2 inhibitor and chemotherapeutic resistance by PAQR8 in breast cancer [118]. Inhibition of PDE4A in breast cancer stem cells increases cAMP levels and PKA activity, which upregulates PTEN and induces cell cycle arrest [119]. The PDE4 inhibitor rolipram synergizes with paclitaxel to inhibit breast cancer cell growth [119]. Meanwhile, PKA-mediated ERK1/2 inhibition may enhance the sensitivity of triple negative breast cancer cells to doxorubicin [121]. cAMP/PKA also inhibits NF-κB signaling in breast cancer thereby suppressing the expression of OCT4, a stem cell marker [122]. It appears that cAMP and PKA have tumor-promoting or tumor-suppressing effects in breast cancer. Given that PTEN deficiency is common in breast cancer, PTEN status may shift the balance between the pro-tumor and anticancer effects of cAMP.

One study suggests that PKA may promote the phosphorylation and nuclear translocation of p53 in breast cancer cells during IL24 treatment [120]. While p53 upregulates cAMP levels in breast cancer cells, it prevents cAMP accumulation in osteoblasts by inhibiting parathyroid hormone-related protein [172]. Therefore, the effects of PKA on p53 are inconclusive or context-dependent. In contrast to the stimulatory effect of PKA on p53 phosphorylation and nuclear translocation in breast cancer cells [120], ADRB1-mediated promotion of cAMP synthesis in BRCA1-deficient ovarian cancer cells suppresses p53 accumulation and DNA damage-induced apoptosis [123]. Similar effect of cAMP/PKA on p53 accumulation was detected in acute lymphoblastic leukemia [173, 174]. In addition, the sympathetic nervous system mediator norepinephrine activates ADRB2 and induces cAMP synthesis, leading to CREB-mediated DUSP1 expression, which dephosphorylates JNK and thereby inhibits c-Jun phosphorylation and ovarian cancer cells apoptosis [124]. Of note, DUSPs may promote the resistance of cancer cells to multiple cancer therapeutic approaches including chemotherapy, radiation and molecular targeted therapy [175]. Except for DUSP1, other cAMP-responsive genes may be involved in cancer therapy. In contrast to DUSP, mitochondrial ferritin is a CREB-regulated gene that can enhance cisplatin sensitivity in ovarian cancer cells [176]. By demethylating m^6^A at the 3'UTR of phosphodiesterases *PDE1C* and *PDE4B*, FTO inhibits *PDE1C* and *PDE4B* expression and thereby enhances cAMP signaling, which suppresses ovarian cancer cells stemness [177].

#### Melanoma

The effects of cAMP signaling are even more complex in melanoma. cAMP stimulates primary melanoma growth through EPAC1/2-Rap1/mTORC1 pathways, while it does not promote metastatic melanoma cells growth [125–127]. Adenylate cyclase inhibitor impairs cAMP signaling and suppresses melanoma growth, while low levels of CREB phosphorylation correlates with melanoma metastasis and recurrence [126]. miR-23a-3p inhibits adenylate cyclase 1 expression and then reduces cAMP synthesis, leading to suppression of mucosal melanoma growth [178]. It is unclear whether the paradoxical effect of CREB on melanoma aggressiveness involves MITF, a CREB-responsive gene that inhibits melanoma invasiveness [179]. In addition, activation of the cAMP-PKA-CREB/CRTC pathway promotes the resistance of melanoma to BRAF(V600E) inhibitor vemurafenib [128]. PKA activity is negatively regulated by PKARIIβ [180, 181]. Both the autophosphorylation site (Ser-116) and the nuclear location signal KKRK are important for the inhibition of PKA by PKARIIβ [180]. Pharmacological activation of PKARII subunits can inhibit proliferation and increased caspase-3 activity in melanoma cells [182].

#### Prostate cancer

PKARIIβ overexpression also leads to prostate cancer cell growth inhibition, whereas overexpression of PKARIA stimulates cell growth [181]. PKA phosphorylates the Thr-89 residue in HSP90 and thereby dissociates HSP90-androgen receptor (AR) complex [129]. In turn, HSP27 binds to the released AR and delivers it into the nucleus [129]. AR-V7, a constitutively active AR variant, induces arginine vasopressin receptor 1a expression in prostate cancer cells, which promotes CREB activation and castration resistance [130]. Treatment of prostate cancer cells with abiraterone acetate increases intracellular cAMP levels and PKA activity, leading to CREB1 phosphorylation that promotes abiraterone acetate resistance [131]. Enzalutamide (MDV3100) treatment also enhances CREB activation in AR-positive prostate cancer cells [132]. CREB can enhance the activity of histone methyltransferase EZH2, which represses the anti-angiogenic factor thrombospondin-1 and promotes neuroendocrine differentiation [132]. Sympathetic activation of cAMP-PKA pathway promotes focal adhesion kinase activation and prostate cancer metastasis [133]. Meanwhile, CREB5 promotes enzalutamide resistance in prostate cancer. In AR-positive prostate cancer cells, CREB5 interacts with and enhances FOXA1 and AR activity thereby regulating a subset of targets such as MYC and genes related to cell cycle, Wnt signaling and EMT [183, 184].

#### Brain tumor

While mutant GNAS drives pancreatic tumorigenesis by promoting PKA-mediated suppression of SIK [185], GNAS acts as a tumor suppressor in neuroblastoma, medulloblastoma and basal cell carcinoma of skin [134, 135, 186]. The development of medulloblastoma and basal cell carcinoma is largely driven by the Sonic Hedgehog (SHH) and Hippo pathways. PKA negatively regulates SHH effectors Smoothened (SMO)-Gli by phosphorylation [187]. Conversely, a PKA-inhibitor motif within SMO physically blocks the active site of PKA catalytic subunit and thereby antagonizes the inhibition of Gli by PKA [188].

In contrast to the tumor suppressive effects of cAMP-PKA signaling in medulloblastoma, the PKA-Dock180 axis mediates the promotion of glioblastoma development and invasion [189]. PKA also phosphorylates glutathione *S*-transferase P1 (GSTP1), an enzyme for carcinogen and drug metabolism, and c-Jun NH_2_-terminal kinase signaling [190]. Phosphorylation of GSTP1 at Ser-184 enhances its enzymatic activity and thereby promotes drug resistance in glioblastoma cells [190]. In addition, PGE_2_-induced cAMP signaling promotes glioblastoma growth, angiogenesis, metastasis and immune evasion [191]. Thus, the cAMP-PKA pathway may have opposing roles in different types of brain tumor.

## cAMP signaling in the tumor microenvironment

The microenvironment of solid tumors is characterized by local hypoxia and higher levels of lactate production. In a panel of carcinoma cell lines of various origin, hypoxia induces adenylate cyclase VI and VII expression through HIF1, which leads to the elevation of cAMP levels and stimulation of cell migration and invasion [192, 193]. Moreover, hypoxia and HIF1α activate PKA by repressing PKARIIβ expression in growth hormone-secreting pituitary tumors [83]. Studies have shown that extracellular lactate can decrease cAMP levels and subsequent PKA activation by binding and activating GPR81 [194, 195]. In murine melanoma cells, lactate increases tumor malignancy by facilitating small extracellular vesicles production via GPR81-cAMP-PKA axis [195]. In the preclinical model of colon cancer, lactate-GPR81-cAMP-PKA signaling promotes chemotherapy resistance through regulating the mismatch repair system [196].

Hypoxia, lactate synthesis and secretion, and overexpression of membrane-bound channels/transporters such as Na^(+)^/H^(+)^ exchanger 1 and bicarbonate symporters in tumor cells may lead to acidic extracellular pH usually ranging from 5.9 to 7.0 [197, 198]. Proton-sensitive GPCRs including GPR4 (GPR19), GPR65, GPR68 and GPR132 are activated by acidic pH via protonation of different histidine residues and other potential acidic residues in these receptors [199, 200]. GPR4 is overexpressed in squamous cell carcinoma of the head and neck, colorectal cancer, breast, ovarian, colon, liver and kidney tumors, Merkel cell carcinoma and melanoma [201–204]. Acidification of the TME is a well-known promoter of tumor progression and immune evasion [205]. Acidic pH stimulates proton-sensitive GPCRs coupling to the Gs protein and thereby induces adenylate cyclase activation and cAMP accumulation [206]. The migration of GPR4 overexpressing melanoma cells is enhanced in an acidic microenvironment [206]. Overexpression of GPR4 in squamous cell carcinoma of the head and neck also induces angiogenesis via IL6, IL8 and VEGFA secretion at acidic extracellular pH [201]. In addition, TGAG8 helps cancer cells adapt to the acidic microenvironment and enforces tumor progression by promoting PKA and ERK activation [207]. Previous studies have demonstrated that TDAG8 and GPR68 are involved in acidic pH-induced expression of programmed cell death protein 1 (PD-L1), an immune checkpoint molecule, in squamous cell carcinoma and melanoma cells [208, 209], which may contribute to immune escape. However, the induction of PD-L1 expression by acidity may be not mediated by cAMP [208].

Cell stress, cell death, and activation of pannexin/connexin channels on endothelial cells and immune cells may lead to the release of ATP into the extracellular space. Extracellular ATP is rapidly degraded to adenosine through two cell-surface ectonucleotidases, CD39 and CD73. Specifically, ATP is converted to ADP and/or AMP by CD39, followed by CD73-mediated dephosphorylation of AMP to adenosine [210]. There are four subtypes of plasma membrane adenosine receptor, including A1R, A2aR, A2bR, and A3R. Similar to PGE_2_ receptor, adenosine receptor couples to specific G protein and activates multiple signaling pathways. Both A2aR and A2bR are coupled to Gαs protein and can induce cAMP synthesis [211], while A1R and A3R inhibit transmembrane adenylate cyclase via Gαi protein, leading to a decrease in cAMP levels.

Accumulating evidence indicates that the neuronal circuits are involved in tumor progression. The infiltration and sprouting of nerve fibers into the tumor microenvironment is a driver of tumor growth and metastasis in various cancer types including prostate, gastric, skin, pancreatic and breast cancer [212–218]. Overexpression of neurotrophic growth factors, such as NGF and brain-derived neurotrophic factor (BDNF), in tumor and stromal cells promotes cancer innervation [213, 215]. Endoplasmic reticulum stress in tumor cells may trigger the expression and release of BDNF into the TME [219]. Given that hypoxia, nutrient shortage and anticancer agents can induce endoplasmic reticulum stress, BDNF expression and release may be induced by diverse stimuli. The sympathetic or parasympathetic nerve fibers in tumor tissue may release neurotransmitters that bind to their receptors in stromal and cancer cells. Catecholamines activates β-adrenergic receptors on tumor or stromal cells and induces cAMP accumulation. In turn, the sympathetic-cAMP-PKA/EPAC signaling pathway promotes tumor growth, invasion and metastasis [133]. β-Adrenergic receptor activation also increases intracellular Ca^2+^, which feedforwards to promote cAMP synthesis [220]. On the other hand, the cAMP/EPAC/JNK signaling pathway promotes BDNF expression in tumor cells, which feedforwards to enhance tumor innervation [221]. Except for stimulation of neurogenesis, BDNF can induce tumor and endothelial cell migration [222]. Collectively, the interplay among neurotransmitters, cAMP and neurotropic growth factors promotes tumor angiogenesis, growth and metastasis [223]. Except for the hypoxic and acidic microenvironment, and the neural niche, tumor stromal cells such as fibroblasts, macrophages and other immune cells are involved in cAMP signaling.

### The roles of cAMP signaling incancer-associated fibroblasts (CAFs)

cAMP/PKA signaling plays an important role in fibroblast cell growth and migration. Inhibition of PKA blocks fibroblast migration in response to serum or PDGF/EGF [224]. Overexpression of RAMP1, a calcitonin gene-related peptide receptor component, can promote mouse skin fibroblast proliferation via Gαi3-PKA-CREB-YAP axis [225]. In human lung fibroblasts, PDE inhibition exerts anti-fibrotic effects via activation of cAMP/PKA signaling and inhibition of TGFβ [226]. Another study reported that PDE4 inhibitors rolipram and roflumilast can antagonize the profibrotic activity of TGFβ1 [227]. Thus, cAMP/PKA signaling is a negative regulator of fibrosis.

Many types of tumor, such as breast, gastric, ovarian, colon, and renal cancer, grow in the anatomical vicinity of adipose tissue. Adipocytes, preadipocytes and adipose tissue support tumorigenesis and metastasis via secreting cytokines and adipokines [228–230]. PKA and EPAC both contribute to the differentiation of 3T3-L1 fibroblast to adipocyte [231]. Moreover, cAMP/PKA/CREB signaling stimulates the adipogenesis of 3T3-L1 by suppressing Dlx5 transcription through upregulated C/EBPβ [232]. These data indicate that cAMP promotes fibroblast differentiation and adipogenesis.

CAFs are abundant components of the TME that have important roles in tumor progression [233]. How does cAMP signaling affect CAF? In breast cancer, estrogen induces FOSL2/Wnt5a expression via cAMP/PKA signaling, in turn, the secreted Wnt5a regulates FZD5/NK-kB/ERK signaling in vascular endothelial cells to promote tumor angiogenesis [234]. Moreover, breast tumor cells could activate ER/GPER/cAMP/PKA/CREB axis in CAFs and trigger the aerobic glycolysis, leading to multiple drug resistance [235]. In gastrointestinal cancers, the expression of GPR68, a proton-sensing GPCR, in CAFs is upregulated by cancer cells [236]. The acidic TME promotes GPR68 activation, cAMP synthesis and PKA activation, leading to increased IL6 expression in CAFs [236]. While CAFs-secreted IL6 appears to reciprocally stimulate tumor cell proliferation [236], sustained IL6 secretion also promotes chronic inflammation and immune evasion [237]. Thus, cAMP-mediated crosstalk between tumor cells and CAFs may promote tumor progression through multiple pathways.

### The roles of cAMP signaling in immune cells

**Fig. 4:**
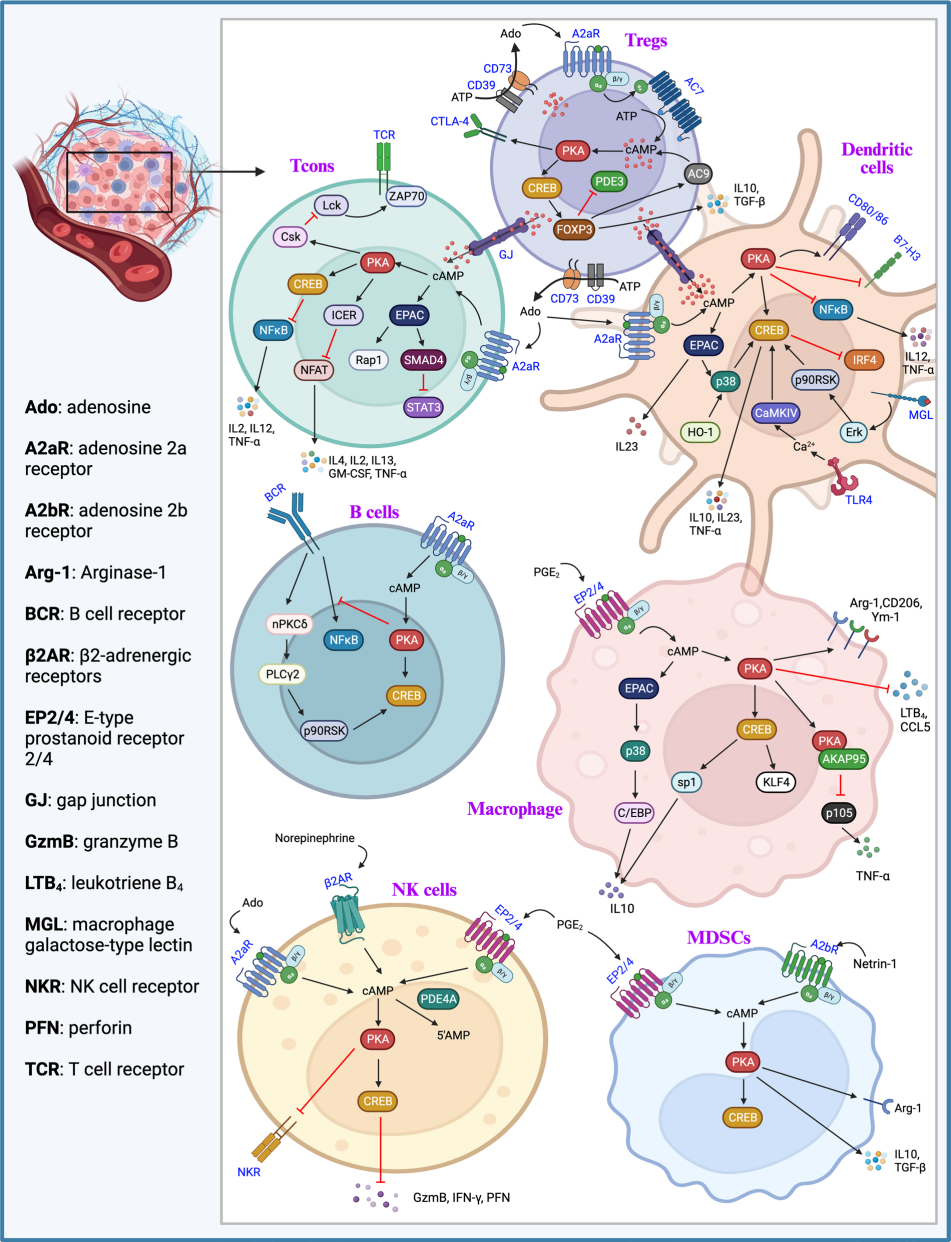
cAMP signaling in immune cells. The GPCR-mediated cAMP signaling is involved in regulating the differentiation, exhaustion and cytotoxic activities of multiple types of immune cells. Both PKA and EPAC mediate the diverse effects of cAMP on immune cells

The immune microenvironment is tightly involved in tumor progression and cancer therapy [238]. Natural killer (NK) cell, macrophage, CD8^+^ effector T cell, CD4^+^ T cell, dendritic cell, T regulator cell, myeloid-derived suppressor cell and B lymphocyte are the building blocks of highly heterogenous tumor immune microenvironment [239]. cAMP has pleiotropic effects on immune cells fate, and may affect immune response (Fig. 4).

#### Regulatory T cells (Tregs)

Many studies have demonstrated the overexpression of CD39, CD73, A2aR, and A2bR in both tumor cells and immune cells, which contributes to extracellular adenosine accumulation, intracellular cAMP production and metabolic reprogramming [240–243]. Besides, the contribution of regulatory T cells (Tregs) to adenosine and PGE_2_ production plays important roles in the TME. Tregs are a small subset of CD4^+^ T cells which interact with effector T cells and suppress their functions [244]. There are two Tregs subtypes, natural Tregs (nTregs) and inducible Tregs (iTregs). iTregs expands and accumulates in tumor tissues and peripheral blood of cancer patients [244]. iTregs expressing both CD39 and CD73 is able to hydrolyze ATP to adenosine. Tr1, a subset of iTregs, is significantly more induced in COX-2-overexpressed tumors than COX-2-negative tumors, while Tr1 cells themselves are COX-2-positive and able to produce and secrete PGE_2_ [245, 246].

Tregs play a central role in the maintenance of self-tolerance and homeostasis through suppressing aberrant immune response against self-antigens, and suppress anti-tumor immune response [244]. The infiltration of Tregs into tumor tissues is associated with poor prognosis in cancer patients [247, 248]. cAMP is crucial for Tregs-mediated immune regulation. The levels of cAMP in Tregs are regulated by the expression and activation of ACs and PDEs. IL2 induces AC7 activation and cAMP accumulation in Tregs [249]. FOXP3, a master regulator of the regulatory pathway in the development and function of Tregs, downregulates miR-142-3p to elevate AC9 expression, resulting in increased cAMP production [250]. Moreover, FOXP3 and miR-142-5p, the predominant miR-142 isoform in Tregs, work in concert to repress PDE3B expression and cAMP degradation in Tregs [250, 251]. Furthermore, PDE4B2 and PDE8A expression are significantly reduced in Tregs as compared with effector T cells [252]. On the other hand, TCR-induced FOXP3 expression in Tregs is controlled by CREB/ATF activation [253]. This FOXP3-cAMP-CREB loop enables Tregs to maintain high levels of cAMP.

The ability of Tregs to generate and accumulate high levels of cAMP enables them to transfer it through gap junction into conventional T cells and dendritic cells [254]. Connexin proteins (Cx)-mediated Gap junction is specialized intercellular channels between two adjacent cells. cAMP induces the expression of Cx43, Cx46, Cx31.1, Cx32 and Cx45 in T cells including Tregs [255]. An alternative mechanism to increase cAMP in the target cells involves the conversion of ATP into adenosine by CD39 and CD73 on the surface of Tregs. Tumor-resident FOXP3^+^ Tregs can decompose ATP to adenosine, which inhibits CD8^+^ T cells proliferation and survival through the A2aR-cAMP pathway [256]. In addition, cAMP signaling in Tregs regulates the expression of other functionally important molecules. cAMP-elevating agents or β2-adrenergic receptor signaling induces the expression of cytotoxic T lymphocyte antigen-4 (CTLA-4), an immune checkpoint molecule, in a PKA-dependent manner [257, 258].

#### Conventional T cells (Tcons)

Tcons include naïve T cells, effector T cells (Teffs), and memory T cells. cAMP plays complex roles in T cell activation, proliferation, and production of cytokines. Adenosine engages A2aR on T cells and thereby activates adenylate cyclase to induce cAMP synthesis [259]. cAMP-PKA activation in Tcons results in the translocation of CREB and CREM/ICER into the nucleus. While CREB activation may promote T cell proliferation and function, such as the production of IFN-γ [260], cAMP-induced expression and nuclear translocation of the transcriptional inhibitor ICER inhibits NF-κB-, AP1-, and NFAT-driven transcription, which in turn impedes the transcription of important genes for immune activation, such as CD25 and IL2, but increases the levels of CTLA-4 [261–264]. In addition, PKA indirectly suppresses Lck and Fyn by phosphorylating Csk at Ser-364, which in turn phosphorylates Lck at Tyr-505 and Fyn at Tyr-528 [265]. The inhibition of Lck/Fyn by Csk prevents zeta-chain-associated protein kinase 70 (ZAP70) phosphorylation, leading to inhibition of TCR signaling [266]. On the other hand, TCR activation by anti-CD3/CD28 stimulation can increase PDE2A and PDE4A/B/D expression in Tcons [267, 268]. Furthermore, evidence suggests that the existence of PKA-independent pathway of cAMP-mediated Tcons suppression. For example, EPAC1 negatively regulates IL2 production and Teffs proliferation in a Rap1-, STAT3- and TGFβ-dependent manner [252, 269]. Selectively activating EPAC1 through 8-pCPT-2’-O-Me-cAMP in mice suppresses CD4^+^CD25^−^ Teffs proliferation [252].

#### Dendritic cells (DCs)

DCs are myeloid cells that have either pro-tumor or anticancer effects [270]. PDE4B is a homeostatic regulator of cAMP in DCs. The dynamic expression of PDE4B is dependent on cAMP/PKA [271]. As the main antigen-presenting cells, DCs are the primary targets of Tregs suppression. The DC-Tregs interaction results in reduced T cell stimulatory capacity and secretion of inhibitory cytokines in DCs. Adenosine induces DCs to migrate toward Tregs through cAMP-PKA-EPAC-Rap1 pathway and then attracts DCs away from CD4^+^ T cells [272, 273]. Furthermore, adenosine-cAMP-PKA/EPAC signaling increases NF-κB expression and immunosuppressive IL10 production, and inhibits IL12p40 production in human monocyte-derived DCs, thereby fully differentiating DCs into a suppressive phenotype [274]. Treatment of DCs with cAMP elevating agent forskolin or co-culture with Tregs leads to a rapid downregulation of co-stimulatory molecules (CD86 and CD80) and upregulation of the inhibitory molecule B7-H3 [275, 276]. Thus, cAMP may reprogram DCs to acquire immune suppressive properties.

The transcriptional activator CREB is a critical regulator of DCs function in immune response. The deletion of CREB in CD11c^+^ cells results in reduced germinal center responses [277]. Heme oxygenase 1 regulates DCs maturation and antigen presentation by modulating p38MAPK-CREB/ATF1 signaling [278]. Independent of cAMP stimulation, DCs also produce IL10 through innate immune receptors via CREB. C-type lectin MGL enhances IL10 and TNFα secretion by DCs via activating ERK-p90RSK-CREB axis [279]. The calcium-dependent CaMKIV-CREB-Bcl2 axis plays an important role in the activation of DCs by TLR4 stimulation [280].

In semi-mature DCs, cAMP upregulates CTLA-2 expression and enhances TGFβ-dependent FOXP3^+^ iTreg conversion [281]. Low levels of cAMP in conventional type-2 dendritic cells (cDC2s) promote Th2 differentiation, while increased levels of cAMP reprogram cDC2s from a pro-Th2 to a pro-Th17 phenotype via repression of IRF4 and KLF4 by the PKA-CREB signaling [282–284]. Accordingly, PDE4 inhibitor augments the Th17-promoting capability of DCs by enhancing IL23 production [285]. Tumor cell-secreted PGE_2_ induces IL23 secretion in DCs via EP2/4-cAMP signaling, leading to Th17 cell expansion [286]. Both PKA-induced phosphorylation of CREB and EPAC-induced phosphorylation of C/EBPβ mediate the stimulatory effect of PGE_2_ on IL23 expression [286]. Th17 cells and Th17-related cytokines have either pro-tumor or anti-tumor roles depending on the cancer type [287, 288]. The exact effects of cAMP-induced Th17-promoting capability of DCs on tumor immune surveillance may be contextual and await further studies.

#### B cells

Except for T cell-mediated cellular immunity, cAMP is also involved in the B cell-mediated humoral immunity. Previous studies have demonstrated that cAMP inhibits B-cell maturation, the activation of quiescent B cells, and subsequent IgG1 and IgE production [289, 290]. In human CD10^+^ B-precursor cells, cAMP elevating agents induce a decline in Mcl1 expression and subsequent apoptosis [291]. Adenosine and cAMP block the B cell antigen receptor-mediated NF-κB activation via PKA [292]. Thus, it appears that cAMP/PKA signaling is detrimental for B-cell maturation and survival. However, CREB is critical for B-cell survival and function. To avoid the detrimental effects of cAMP and PKA on B cells, mature B cells take advantage of PKCδ and p90RSK rather than PKA to activate CREB, which contributes to the regulation of numerous CRE-dependent genes involved in B-cell function [293]. Tumor necrosis factor receptor-associated factor 3 (TRAF3) plays a critical role in inhibiting B-cell survival via inhibiting CREB stability [294]. Loss-of-function mutations of TRAF3 are commonly found in multiple myeloma, ovarian cancer and B-cell lymphoma [295, 296].

#### Macrophages

While phagocytosis of tumor cells by macrophages is a mechanism of immune surveillance, the tumor-associated macrophage (TAM) is a specific subpopulation of macrophages within the TME that are reprogrammed and hijacked by tumor cells to promote tumor progression [297]. cAMP has important roles in macrophage functions such as phagocytosis and microbial killing [298]. In alveolar macrophages, the resident immune effector cells in the lung, EPAC1 activation restrains phagocytosis, while PKA activation suppresses the production of leukotriene B_4_ (LTB_4_) and TNFα [299]. It is also reported that cAMP inhibits LPS-induced TNFα secretion via PKA-AKAP95-NFkBp105 in murine macrophage RAW264.7 cells [300]. cAMP and PGE_2_ stimulate the production of IL10 and G-CSF through PKA in RAW264.7 cells, which induces a pro-tumorigenic macrophage phenotype [300–303]. PGE_2_ secreted from breast cancer cells suppresses CCL5 secretion in LPS-activated macrophages through cAMP/PKA signaling pathway [304]. Moreover, cAMP signaling stimulates the M2 polarization of macrophages through a PKA-C/EBPβ-CREB dependent pathway in murine macrophages [305]. PGE2 enhances M2 polarization via the CREB-mediated induction of KLF4 [306]. Db-cAMP, a cAMP mimetic, promotes reprogramming of bone-marrow-derived macrophages to a M2 phenotype through increasing Arg-1/CD206/Ym-1 expression and IL10 levels in a PKA-dependent manner [307]. β2-Adrenoceptors in tumor-associated macrophages contribute to HCC progression through activating cAMP/PKA/CREB and cAMP/IL6 signaling pathways [308]. Thus, PKA is tightly involved in the promotion of pro-tumor macrophage polarization.

#### NK cells

NK cells are immune effectors that can directly recognize and kill tumor cells [309]. The presence of immunosuppressive factors in the TME, including PGE_2_, adenosine and cAMP, limits tumor-infiltrating NK cells persistence. cAMP signaling modulates the cytotoxicity of NK cells. When NK cells are exposed to lysis-sensitive tumor target cells, there is an increase in intracellular cAMP in NK cells, but no increase in response to lysis-resistant tumor target cells [310]. Several studies have identified that cAMP can inhibit the cytotoxicity and secretion of granzyme B, perforin and IFN-γ from NK cells through PKA/CREB signaling [311–315].

Recently, a study has shown that PDE4A confers resistance to PGE_2_-mediated suppression in NK cells by reducing intracellular levels of cAMP [316]. Moreover, adenosine suppresses various cytokines/chemokines production and inhibits the cytotoxic activity of human and murine NK cells via stimulation of A2aR/AC/cAMP signaling and subsequently activation of PKA type I [313, 317]. Blocking regulatory, but not catalytic, subunits of PKA type I abrogates the inhibitory effects of adenosine [313]. These data suggest that the regulatory subunits of PKA type I are the prominent contributor to the cAMP-mediated inhibitory effect on NK cells.

#### Myeloid-derived suppressor cells (MDSCs)

MDSCs are natural immunosuppressive cells, which block adaptive immunity by inhibiting the activation of CD4^+^ and CD8^+^ T cells and suppress innate immunity by inhibiting NK cells [318, 319]. PGE_2_ can induces COX_2_ expression in cultured peripheral blood-isolated monocytes, blocking their differentiation into CD1a^+^ DCs and promoting their development into MDSCs [320]. In a 4T1 mammary carcinoma model, PGE_2_ promoted tumor progression by inducing MDSCs partially through the EP2 receptor [321]. In addition to PGE_2,_ selective EP2 and EP4 agonists, but not EP1/3 agonists, also induce MDSCs development through the AC/cAMP/PKA/CREB signaling pathway [320, 321]. MDSCs play a prominent role in tumor progression [322]. In a B16F10 mouse melanoma model, mice treated with a selective A2bR agonist Bay60-6583 showed increased VEGF production from MDSCs and tumor vessel density [323]. Pharmacological blockade of A2bR with PSB1115 reduced tumor-infiltrating MDSCs and restored an efficient antitumor T cell response, leading to a significant suppression of melanoma growth [323, 324]. Given that MDSCs has emerged as important contributor to tumor progression, more powerful evidence is needed to determine whether cAMP signaling can directly induce the development and activation of MDSCs.

## Targeting cAMP signaling for cancer therapy

### PKA inhibitor

Since the cAMP-PKA pathway is pro-tumorigenic and immunosuppressive in many types of cancer, PKA inhibitors may have anticancer effects in some types of cancer. There are three types of PKA inhibitor, including cAMP analog, small-molecule PKA inhibitor, and peptide inhibitor (Table 2). cAMP analogs have been shown to inhibit PKA activity by binding to the ATP-binding pocket of the PKA regulatory subunit [325]. Rp-8-Br-cAMP is a commonly used cAMP analog that competes with endogenous cAMP to bind to the PKA regulatory subunit. H89 is a widely used PKA inhibitor in preclinical studies, while it is non-specific. Treatment with H89 suppressed small cell lung cancer and Ewing sarcoma progression in animal models [326, 327]. Given that the PKA catalytic subunit also promotes the immunosuppressive macrophage phenotype in tumors, inhibition of PKA may have immunotherapeutic effects, especially when combined with αCTLA-4 antibody [328]. Liposomal H89 complexes with a diameter of about 1000 nm can be taken up by cancer-associated macrophages and thereby inhibit tumor growth and metastasis by promoting T cell activation [328]. KT-5720 is another PKA inhibitor with tumor suppressive effects. The MDR1-mediated drug resistance in hematological malignancies could be reversed by KT-5720 [329]. Daphnetin, a dehydroxylated derivative of coumarin isolated from plants Daphne species, has PKA-inhibitory activity with an IC50 value of 9.33 µM; however, it also non-specifically inhibits EGFR and PKC [330, 331]. Preclinical studies demonstrate that daphnetin has anticancer potential against leukemia, osteosarcoma, breast, ovary, kidney, colon, and liver cancers [332–337]. Recently, a novel diarylcyclohexanone derivative MHY4571 was developed as an orally active PKA inhibitor with anticancer effects [11].

**Table 2:** List of the compounds targeting cAMP signaling

Category	Target	Antagonist	Agonist
cAMP synthesis modulator	Adenylate cyclase	SQ22536; NB-001; ST034307; AC10065; MDL-12,330A	Forskolin
	sAC	Bithionol; Hexachlorophene; KH7; TDI-10229; TDI-11861; LRE1	
Regulator of the cAMP sensor	PKA	^a^KT5720; H89; GSK299115A; GSK466317A; STAD 2; HA-1004; Daphnetin; MHY4571	6-Bnz-cAMP; 8-Bromo-cAMP; Sp-cAMPS; CW 008; Bucladesine
		^b^Rp-8-Br-cAMP; Rp-cAMPS	
		^c^PKI(5-24)amide; PKI(5-22)amide; PKI-(6-22)-amide; PKI(14-22)amide; PKI(14-24)amide; Malantide	
	Pan-EPAC	ESI-08; ESI-09; HJC0197	8-CPT-Cyclic AMP; 8-pCPT-2'-O-Me-cAMP***-***AM
	EPAC1	AM-001; CE3F4; EPAC 5376753	I942; SY009
	EPAC2	AAK-399; AAD-026; MAY0132; HJC0350	
Regulator of cAMP/cGMP hydrolysis	PDE3	Cilostamide; Cilostazol; Olprinone; K134	
	PDE4	Rolipram; Eggmanone; Roflumilast; Cilomilast; Mufemilast; Zatolmilast; Oglemilast; Tetomilast; Lotamilast; ML-030; VI-1004; MR-L2; YM976; MK-0952; GSK356278; GSK256006; PF-06445974; D159687	
	PDE5	Sildenafil; Vardenafil; Tadalafil; Avanafil; FR-229934	

^a^Small-molecular inhibitor

^b^cAMP analog

^c^Peptide inhibitor

Protein kinase inhibitor peptide (PKI) is another category of specific PKA inhibitor (Table 2). The endogenous PKI isoforms, PKIα/β/γ, are negative regulators of PKA activity. Several synthetic PKA inhibitor peptides have been developed. Among these peptides, PKI-(6-22)-amide is the most potent PKA inhibitor, followed by PKI-(5-24)-amide and PKI-(14-24)-amide. For detailed information on the development of PKA inhibitor peptides, we refer readers to the review by Liu et al. [325].

### EPAC inhibitor

PKA can negatively feedback to inhibit cAMP synthesis. Thus, PKA inhibition may result in an increase in cAMP levels and divert cAMP signaling toward EPAC [338]. While EPAC activation may be detrimental to tumor suppression, H89 synergizes with the oncolytic virus M1 to inhibit tumor growth through EPAC activation [339]. Hence, this seemingly adverse effect may be exploited for combined therapy. It warrants further studies to determine the effects of 8-CPT-cAMP and 8-pCPT-2'-O-Me-cAMP***-***AM, two EPAC agonists, in tumor progression. In addition, I942 and SY009 are two EPAC1-specific non-cyclic nucleotide agonists which effects in cancer are poorly studied [340, 341].

3-(5-tert-butyl-isoxazol-3-yl)-2-[(3-chloro-phenyl)-hydrazono]-3-oxo-propionitrile (ESI-09) is a non-cyclic nucleotide EPAC-selective inhibitor that suppresses Rap1 activation, Akt phosphorylation and pancreatic cancer cell invasion [342, 343]. Other pan-EPAC inhibitors include ESI-08 and HJC0197. Further studies develop EPAC1- or EPAC2-specific inhibitors. While AAK-399, AAD-026 and MAY0132 are EPAC2-specific inhibitors [344], AM-001 and the (R)-enantiomer of CE3F4 (R)-CE3F4 are potent EPAC1 antagonists [345, 346]. So far, there are little reports on the effects of these EPAC inhibitors on tumor therapy. The inhibition of insulin secretion by AAK-399 and AAD-026 may be an adverse effect [344].

### Adenylate cyclase and sAC inhibitors

Instead of inhibiting PKA and EPAC, another strategy to target cAMP signaling is direct inhibition of cAMP synthesis. SQ22536 (9-(tetrahydro-2-furanyl)-9H-purin-6-amine) is an adenine-like adenylate cyclase inhibitor. Another adenylate cyclase 1 inhibitor, NB-001, has analgesic effect on cancer pain [347]. The safety of NB001 is being tested in human clinical trials [348]. While the chromone-based ST034307 selectively inhibits adenylate cyclase 1 [349], the oxadiazole-based AC10065 can suppress both adenylate cyclase 1 and adenylate cyclase 8 [350]. The adenylate cyclase inhibitor MDL-12,330A suppresses cAMP synthesis in tumor tissue, leading to immune system-dependent inhibition of tumor progression [351]. In addition, the GPCR-adenylate cyclase-cAMP-PKA-CREB pathway is involved in the resistance of melanoma to MAPK inhibitors [352]. Except for the transmembrane adenylate cyclase, sAC is another target for cancer therapy. Bithionol and hexachlorophene are potent sAC-specific inhibitors that bind to bicarbonate-binding site [353]. Bithionol has been shown to enhance the sensitivity of ovarian cancer cells to cisplatin and paclitaxel [354]. These sAC inhibitors can suppress mitochondrial respiration and thereby limit the energy source in tumor cells [355].

Chimeric antigen receptor (CAR) T-cell therapy, an adoptive T-cell therapy involving the ex vivo transduction of a patient’s T cells with an engineered CAR that targets a defined tumor antigen, is a promising immunotherapy for hematological malignancies [356]. Since the adenosine-A2aR-adenylate cyclase-cAMP axis suppresses the function of CD8^+^ T-cell as well as CAR T-cell [357], blockade of A2aR in CAR-T cells can improve the efficacy of adoptively transferred T cells [358, 359]. It remains to know whether blockade of the predominant adenylate cyclase isoforms in CAR T-cells can achieve similar effect. Currently, the efficacy of CAR T-cell therapy is relatively poor in solid tumors [360], targeting the A2aR-adenylate cyclase-cAMP axis in CAR T-cells holds promise in improving CAR T-cell therapy for solid tumors.

### cAMP-elevating agents

While inhibition of cAMP synthesis or PKA/EPAC can inhibit some types of cancer and stimulate anticancer immunity, cAMP-elevating agents such as the adenylate cyclase agonist forskolin and the cAMP analog 8-Br-cAMP also emerge as potential cancer therapeutics, especially when combined with other anticancer agents. Forskolin enhances paclitaxel- and H3K27me2/3 demethylases inhibitor GSKJ4-induced cytotoxicity in non-small-cell lung cancer and acute myeloid leukemia cells [95, 361]. Co-delivery of paclitaxel and forskolin by liquid crystal nanoparticles can inhibit the stemness of breast cancer stem cells and reverse chemoresistance, thereby eliciting potent antitumor activity [362]. Given the favorable safety profile, forskolin has been used to treat patients with glaucoma, asthma and heart failure [363]. However, the poor pharmacokinetic profile may make forskolin less effective for cancer therapy. The clearance rate and half-life for forskolin are 0.53 L/h and 3.9 h in mice, respectively [362]. It may be necessary to develop new delivery system to improve the efficacy of forskolin. cAMP analogs also inhibit medullary thyroid cancer cell growth [364]. PKA-mediated inhibitory phosphorylation of Raf1 contributes to the anticancer effects of cAMP, while cAMP activators also induce p21 expression independent of PKA. 5-Demethylnobiletin, a natural polymethoxyflavone in the extract of citrus fruits peels, is able to stimulate cAMP signaling and inhibit cancer [365, 366]. Nevertheless, these natural agents usually have multiple targets.

### PDE inhibitors

An alternative choice to elevate intracellular cAMP levels is the suppression of PDE. Isobutylmethylxanthine is an anticancer agent that inhibits PDE3/4/5 with the IC50 values ranging from 6.5 to 31.7 µM [367]. Cilostamide and cilostazol are PDE3 inhibitors that have tumor suppressive activity in both solid tumors and hematological malignancies. Cilostamide synergizes with imatinib to inhibit cancer [368]. In humans receiving single oral administration of cilostazol at dose of 100 mg, the mean maximum concentration in serum (Cmax), concentration–time curve (AUC) and half-life (T_1/2_) are 701 ng/ml, 13,724 ng × h/mL and 13.5 h, respectively (https://drugs.ncats.io/drug/N7Z035406B). Combined treatment with statin and cilostazol can inhibit AML and MM cells survival [369]. In addition, cAMP can induce diffuse large B-cell lymphoma cell apoptosis by inhibiting spleen tyrosine kinase (SYK)/PI3K/AKT pathway, which is independent of PKA and EPAC [96]. Many receptor or non-receptor tyrosine kinases are the targets of cancer therapy. FDA-approved SYK inhibitors have been used to treat cancer [370]. The inhibition of SYK by cAMP may underlie the anticancer effects of certain agents that elevate intracellular cAMP levels. While PKA reportedly inhibits the activity of SYK in neutrophils [371], it promotes SYK activation in platelet [372]. It remains unclear how cAMP inhibits SYK in diffuse large B-cell lymphoma cell. Further studies are needed to determine the role of PKA in the regulation of SYK by cAMP in diffuse large B-cell lymphoma cell.

Rolipram is an inhibitor of PDE4A, PDE4B and PDE4D with its IC50 values of 4, 20 and 33 nM, respectively. Rolipram and another PDE4 inhibitor, eggmanone, suppress SHH and Hedgehog signaling thereby inhibiting medulloblastoma growth in GNAS-mutant mice [134, 373, 374]. PDE4A and PDE4D promote HIF signaling in lung cancer through cAMP-PKA/EPAC pathways. Treatment of lung cancer with PDE4 inhibitor suppresses cancer cell growth and angiogenesis [375]. PDE4D is overexpressed in LKB1-mutated lung cancer; therefore, PDE4 inhibitors may be more effective in treating LKB1-mutated lung cancer [376]. In addition, the PDE4D inhibitor roflumilast suppresses the growth of medulloblastoma that is resistant to SHH antagonist vismodegib [377]. Given that PDE4D interacts with mTORC1 and promotes its activation by inhibiting PKA-mediated raptor phosphorylation at Ser-791, pharmacological inhibition of PDE4D by roflumilast or GEBR-7b suppresses pancreatic cancer cell growth in vitro and in vivo [16]. Pharmacologic inhibition of PDE4D also suppresses prostate cancer growth by blocking sonic hedgehog, androgen receptor and MAPK pathways, and reverse tamoxifen resistance in estrogen receptor-positive breast cancer [378, 379]. Moreover, adenylate cyclase activator in combination with PDE inhibitor more significantly elevates cAMP levels and overcomes chemoresistance. Forskolin together with rolipram potently inhibits chemoresistant colon cancer cell growth [380]. PDE4B is overexpressed in diffuse large B-cell lymphoma and prevents cAMP-induced apoptosis [96, 381]. Hence, PDE4B inhibitors may have tumor suppressive effects in diffuse large B-cell lymphoma. PDE4B also restricts cAMP levels in colon cancer and abrogates cAMP-induced anticancer effect [382]. These studies indicate that PDE4 inhibitors may have anticancer effects on various types of cancer with specific genetic background.

PDE5 usually controls the degradation of the second messenger cGMP, an activator of protein kinase G (PKG); however, cGMP has been shown to inhibit the degradation of cAMP by other PDE family members [383]. Thus, the PDE5 inhibitors may indirectly elevate cAMP levels in tumor cells. Many PDE5 inhibitors such as sildenafil, vardenafil, tadalafil, and avanafil have been approved to treat pulmonary arterial hypertension. PDE5 inhibition also eliminates cancer stem cells, possibly through cAMP-PKA signaling [384]. Sildenafil, tadalafil and vardenafil impact cAMP-specific PDE8 isoforms-linked second messengers and steroid production in a mouse Leydig tumor cell line [383]. Accumulating evidence demonstrates that PDE5 inhibitors have anticancer effects by synergizing with chemotherapeutic agents [385–387]. Sildenafil and vardenafil have been shown to inhibit B-cell chronic lymphocytic leukemia cells growth [384]. Treatment of cancer cells with sildenafil increases the sensitivity of other chemotherapeutic drugs such as vincristine, etoposide, doxorubicin and cisplatin [388].

Some natural agents can also inhibit PDEs. Luteolin and 6-gingerol are PDE inhibitors from artichoke (*Cynara scolymus*) and ginger (*Zingiber officinale*), respectively [389]. In addition, the antimalarial agent artemisinin can inhibit calmodulin-mediated activation of PDEs and elevate cAMP levels [390]. These phytochemicals have anticancer effects especially when combining with other cytotoxic drugs [391–394]. Since these agents have multiple targets, it remains to know how cAMP may be involved in the anticancer effects of these agents.

## Clinical development of anticancer agents targeting cAMP signaling pathways

Although PKA inhibitors can suppress tumor progression in some animal models, there are no PKA inhibitors undergoing clinical trials for cancer therapy. Global inhibition of cAMP, PKA and EPAC is challenging, due to the ubiquitous expression and activity of PKA and EPAC in normal physiology such as heart rhythm, optimum cardiac performance, synaptic plasticity, insulin secretion, sleep, learning and memory [395–398]. In addition, the pharmacological PKA inhibitors H89 and KT5720 have widespread effects independent of PKA [399]. Therefore, these inhibitors have on-target and off-target side effects. Some of the adverse effects, such as anxiety and depression disorders, may be intolerable [400]. To enable safe administration of PKA inhibitors for cancer therapy, future efforts may be made to precisely deliver PKA inhibitors to tumor site.

While the progression of PKA inhibitors into the clinic has been held back by severe adverse effects, many PDE inhibitors have been used in clinical setting. The PDE4 inhibitors roflumilast (Daliresp, Daxas), apremilast (Otezla), and crisaborole (Eucrisa) are in the market for treating chronic obstructive pulmonary disease, psoriasis, and moderate atopic dermatitis, respectively [401]. There are some clinical trials aiming to evaluate the safety and efficacy of PDE inhibitors in cancer patients (Table 3). In humans receiving single oral administration of roflumilast at a dose of 0.5 mg, the Cmax, AUC and T_1/2_ are 12.5 ng/ml, 65.1 ng × h/mL and 19.9 h, respectively (https://drugs.ncats.io/drug/0P6C6ZOP5U). Roflumilast in combination with prednisone was safe in patients with advanced B-cell malignancies; in this small-scale clinical trial, combination of roflumilast and prednisone resulted in partial response or stable disease in 66% of patients [402]. It remains to know the outcome of this regimen in larger clinical trials. The plasma concentration of roflumilast levels can reach 8.2 ng/ml, which is markedly above the IC50 value for inhibition of PDE4 (0.8 nM) [402]. In another pilot clinical study on relapsed/refractory diffuse large B-cell lymphoma (DLBCL), roflumilast in combination with etoposide, cisplatin, methylprednisolone and cytarabine resulted in better CR (46.2% vs. 34.6%), ORR (76.9% vs. 53.8%), and 1-year PFS (50.0% vs. 25.9%) compared with the chemotherapy-alone group [403]. There was no difference in median overall survival and 1-year overall survival between the roflumilast and chemotherapy-only arms. Importantly, PDE4B was a key downstream effector of DPY30, and the PDE4 inhibitor rolipram preferentially targeted DPY30-expressing cells [404]. It remains to know whether DPY30 can serve as a biomarker for precision treatment with PDE4 inhibitor such as roflumilast.

**Table 3:** Clinical trials of PDE inhibitors for cancer therapy (ClinicalTrials.gov)

Trial ID	Regimen	Target	Cancer type	Phase	Outcome	References
NCT01888952	**Roflumilast** Prednisone	PDE4	B-cell lymphoid malignancies	I	66% patients had PR or SD	[402]
NCT03458546	**Roflumilast** R-CHOP	PDE4	Diffuse large B-cell lymphoma	I	NA	
NCT05796271	**Roflumilast** R-CHOP	PDE4	Diffuse large B-cell lymphoma	I	NA	
NCT02544880	**Tadalafil** Anti-MUC1 vaccine	PDE5	Head and neck squamous cell carcinoma	I	The treatment combination is safe and well-tolerated	[405]
NCT03238365	**Tadalafil** **Nivolumab**	PDE5	Head and neck squamous cell carcinoma	I	T lymphocyte and myeloid cell infiltration is enhanced	[406]
NCT02466802	**Sildenafil** Regorafenib	PDE5	Solid tumors	I	Regorafenib can be safely combined with sildenafil	[407]
NCT02279992	**Vardenafil** Carboplatin	PDE5	Gliomas and brain metastases	I	NA	
NCT03993353	**Tadalafil** Pembrolizumab	PDE5	Head and neck cancer	II	NA	
NCT01950923	**Sildenafil**	PDE5	Kidney cancer	I	NA	
NCT05014776	**Tadalafil**	PDE5	Pancreatic cancer	II	NA	
NCT03785210	**Tadalafil** Nivolumab vancomycin	PDE5	HCC and liver metastases from colorectal cancer or pancreatic ductal adenocarcinoma	II	OS: 9.4 m in HCC group; 3.4 m in liver metastases group	
NCT04069936	**Tadalafil** Nivolumab MILs™	PDE5	Non-small-cell lung cancer	II	NA	
NCT01817751	**Tadalafil** Sorafenib Valproic acid	PDE5	Glioma	II	6.4% of participants met 12-m PFS	
NCT00843635	**Tadalafil**	PDE5	Cancer of the oral cavity or oropharynx	Not applicable	NA	
NCT00752115	**Sildenafil** Paclitaxel Carboplatin	PDE5	Non-small-cell lung cancer	II/III	NA	
NCT05709574	**Tadalafil** Neoadjuvant FLOT	PDE5	Gastric/gastroesophageal junction adenocarcinoma	II	NA	
NCT01697800	**Tadalafil**	PDE5	Head and neck squamous cell carcinoma	II	NA	

FLOT: fluorouracil, leucovorin, oxaliplatin, and docetaxel; MILs™: marrow infiltrating lymphocytes; NA: not available; OS: overall survival; PFS: progression-free survival; PR: partial response; R-CHOP: rituximab plus cyclophosphamide, doxorubicin, vincristine, and prednisone; SD: stable disease

While the association between PDE5 inhibitors and colorectal cancer risk is controversial [408, 409], one study indicates that the use of PDE5 inhibitor after surgical resection may be related to a reduced risk of colorectal cancer-specific mortality [410]. Exisulind is a sulindac metabolite without anti-inflammatory activity, while it inhibits PDE5 [411]. One clinical trial has evaluated the effectiveness and safety of PDE5 inhibitors in treating metastatic breast cancer. Treatment of breast cancer patients with exisulind and capecitabine was well tolerated, while the effectiveness may be limited at the tested dosage [412]. Another phase I clinic trial explored the pharmacokinetic profile of exisulind in patients with advanced solid tumors [413]. There was a significant correlation between the plasma concentrations of exisulind and gastrointestinal toxicities [413]. In addition, clinical trials demonstrated that tadalafil increased T-cell expansion, reduced peripheral MDSC and Tregs numbers, and enhanced tumor-specific immunity in response to head and neck squamous cell carcinoma (HNSCC) lysate [414, 415]. Another clinical trial of combining tadalafil with the MUC1/polyICLC vaccine also demonstrates the safety and immunologic potential of this regimen in HNSCC patients [405]. However, the expression of PDL1 is upregulated after the treatment, indicating additional immune evasion [405]. A neoadjuvant randomized trial in patients with resectable HNSCC demonstrates that combination of the PDE5 inhibitor tadalafil with the PD-1 inhibitor nivolumab is safe and enhances immune-mediated anticancer effects, leading to pathologic treatment response in more than 20% patients [406]. Sildenafil, another PDE5 inhibitor that has been used in the clinic. A phase I trial has been conducted to evaluate the combination of sildenafil and regorafenib, a multi-kinase inhibitor, in advanced solid tumors [407]. Sildenafil can increase intratumoral T cell infiltration and activation, thereby improving the anticancer effectiveness of adoptive T cell treatment. More clinical studies are needed to evaluate the anticancer effects of PDE inhibition in combination with immunotherapy.

## Concluding remarks

In conclusion, cAMP and the tumor microenvironment play a complex and interdependent role in cancer progression. cAMP may promote or inhibit cancer cell growth and metastasis in cancer types- and context-dependent manners. Activation of PKA by cAMP may either enhance or inhibit the activity of different oncogenes or tumor suppressors. Therefore, the type of oncogenic alteration is a determinant of the complex roles for cAMP signaling in cancer. Tumors with the *DNAJB1*–*PRKACA* gene fusion or *BAP1*-mutation share a molecular hallmark, namely aberrant activation of PKA [416]. Certainly, there are other driving events that lead to hyperactivation of cAMP signaling pathways during the development of some types of cancer. While cAMP promotes tumors such as FLC and basal cell carcinoma, it functions as a tumor suppressor in medulloblastoma by inactivating Gli. In addition, *BRAF* mutation may override the inhibition of Raf1 by PKA and thereby shift the balance between the tumor-promoting and tumor-suppressing effects of cAMP.

Although selective inhibitor of PKA can suppress the growth of PKA-addictive tumors, clinical development of such an inhibitor remains to be highly problematic because of the toxicity resulting from inhibition of the critical physiological functions of normal PKA. The discovery of selective small-molecule inhibitors of the *DNAJB1*–*PRKACA* chimera would be wonderful for precise treatment. Future efforts aimed at tumor-targeted delivery of PKA inhibitor may help break these bottlenecks. Alternatively, understanding the precise mechanisms by which cAMP, PKA and other effectors interact with the tumor microenvironment to promote tumor progression and immune resistance will be helpful for the development of new and effective therapies that target the key nodes in cAMP signaling. Evidence indicates that inhibition of cAMP synthesis can restore antitumor immunity and suppress tumorigenesis [351]. Except for the immune checkpoints, the RIG-I-like receptors/MAVS signaling pathway is emerging as another target for cancer immunotherapy [417]. cAMP/PKA promotes MAVS degradation by phosphorylating it at T54 [418]. It remains to know whether PKA inhibitors can improve the RIG-I-like receptors-targeted immunotherapy.

cAMP has both proapoptotic and antiapoptotic effects [44]. Currently, more efforts are paid for exploring the efficacy of cAMP-elevating compounds on cancer therapy. Indeed, cAMP and its signaling transducers such as PKA not only upregulate many oncogenes but also inactivate some oncogenes. Some types of cancer, such as basal cell carcinoma and medulloblastoma, are driven by oncogenes that are suppressed by cAMP and PKA. Therefore, elevating cAMP levels is supposed to be effective in treating these types of cancer. PDE inhibitors hold promise for cancer therapy. Many PDE inhibitors have been safely used for treating different diseases, and can be repurposed for cancer therapy. While PKA can inhibit cell apoptosis, it paradoxically promotes caspase-independent parthanatos, a type of programmable cell death characteristic of chromatinolysis and large-size DNA fragmentation, by phosphorylating PARP1 [47]. Also, the activation of EPAC may contribute to parthanatos by inhibiting PARP cleavage and upregulating PARP expression [419]. Thus, PDE inhibitors may synergize with agents that induce parthanatos. In addition, activation of PKA and EPAC by cAMP may sensitize cancer cells to lysosome-dependent cell death. PDE inhibitors in combination with lysosome-destabilizing agent may synergistically induce cancer cell death. Together, both tumor types and the exact nature of combined agents should be taken into consideration when cAMP-elevating compounds are supplemented with other anticancer agents. Another critical concern for the administration of cAMP-elevating agents for cancer therapy is the immune-suppressive effects of cAMP. Nonetheless, recent study indicates that the PDE4 inhibitor roflumilast does not reduce the clinical activity of immune checkpoint inhibitors, the mainstay in cancer immunotherapy [420, 421]. It warrants further studies to confirm this issue. Given that ferroptosis is inhibited by PKA but promoted by EPAC, PKA inhibition may enhance the efficacy of cAMP-elevating compound in combination with ferroptosis-inducing agents.

## Abbreviations

AC: Adenylate cyclase
AKAP: A kinase anchor protein
CAF: Cancer-associated fibroblast
cAMP: Cyclic adenosine monophosphate
CREB: cAMP-responsive element-binding protein
DCs: Dendritic cells
EBV: Epstein–Barr virus
EPAC: Exchange protein activated by cAMP
FLC: Fibrolamellar hepatocellular carcinoma
GPCR: G protein-coupled receptor
MDSC: Myeloid-derived suppressor cell
NETs: Neutrophil extracellular traps
PDE: Phosphodiesterase
PKA: cAMP-dependent protein kinase
PKI: Protein kinase inhibitor peptide
TAM: Tumor-associated macrophage
TME: Tumor microenvironment
